# A case report of triple neural tube defect: revisiting the multisite closure theory

**DOI:** 10.1186/s12893-019-0633-2

**Published:** 2019-11-06

**Authors:** Jayant Kumar Yadav, Ahtesham Khizar, Pradhumna Kumar Yadav, Ghulam Mustafa, Sajid Nazir Bhatti

**Affiliations:** 1 Tribhuvan University Teaching Hospital, Maharajgunj, Kathmandu, Nepal; 2 Pakistan Institute of Medical Sciences, Islamabad, Pakistan

**Keywords:** Triple neural tube defect, Multisite closure theory, Encephalocele, Meningomyelocele, Neurulation

## Abstract

**Background:**

Triple neural tube defects are rare. To the author’s knowledge, there are only four reported cases available in the literature up to date. Controversies exist with regards to the development of neural tube defects. We revisit the multisite closure theory and try to explain the mechanism of neural tube defects in our case.

**Case presentation:**

We report a case of one-month-old baby boy who presented to us with three distinct neural tube defects. He had occipital and cervical encephaloceles along with thoracolumbar myelomeningocele accompanied by syrinx and mild hydrocephalus. All the three defects were surgically corrected with good neurological outcome.

**Conclusion:**

In the multisite model of human neural tube closure, there are only two fusion sites and two neuropores unlike in mouse. This can explain the origin of open neural tube defects including anencephaly and myelomeningocele (as in our case) but cannot account for the development of encephalocele, which appears to be a post neurulation defect.

## Background

Triple neural tube defect (NTD) is a rare entity with only four cases previously reported in the literature [1–3]. NTDs arise due to the failure of the neural plate to oppose into folds and fuse during neurulation. The type and severity of these NTDs varies depending on the level of body axis affected. The pathogenesis underlying the neural tube defect is very complex involving complex interaction between genes, environment and nutrition [4]. Several hypotheses have been proposed to explain neural tube development based on the experimental models. This is because neurulation stage human embryos have been rarely available. We revisit the multisite closure theory to explain the findings in our case based on the observation from human embryos.

## Case presentation

**Fig. 1:**
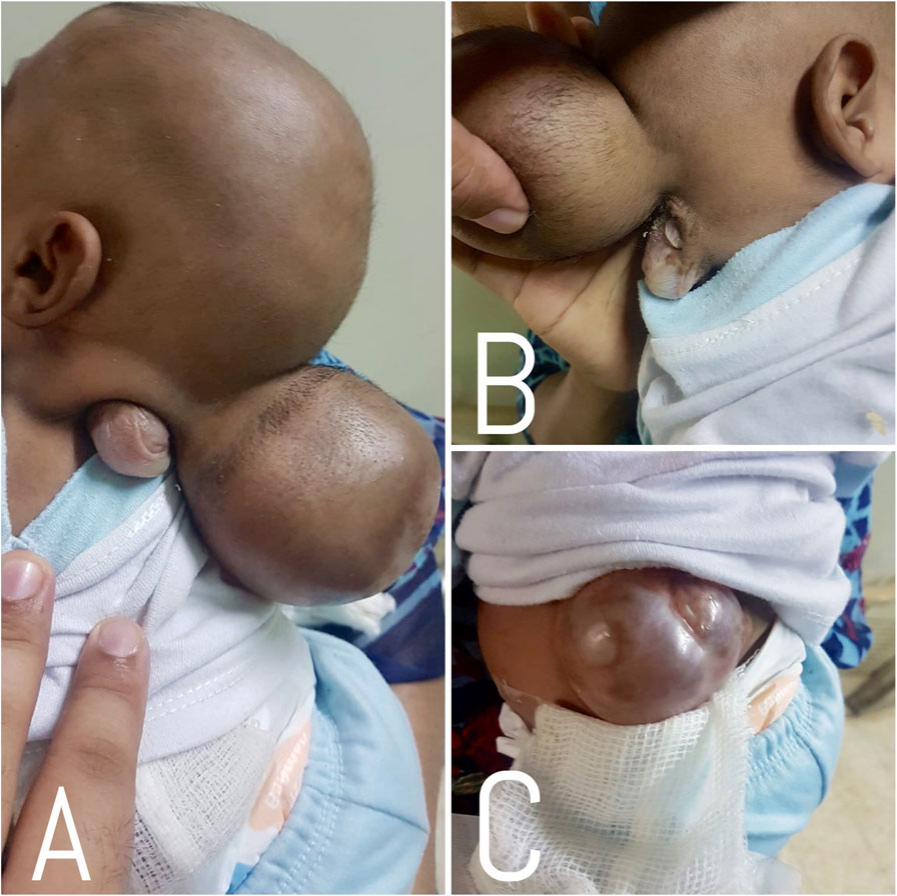
Preoperative **a** & **b** Occipital & Cervical Encephaloceles, **c** Thoracolumbar Meningomyelocele

A 1 month old baby boy from rural Pakistan presented to a university hospital with three swellings- two swellings in the occiput and cervical region and third swelling in the thoracolumbar region. His mother informed us that the swellings were present at the time of birth. Besides, these swellings, the mother also complained that the baby moved both his lower limbs sluggishly. The baby was delivered vaginally at 36 weeks of gestation at a local hospital and the baby cried immediately after birth. She had not received any antenatal care. On examination, there were two cystic swellings on the occipital and cervical region each measuring 9 × 6 cm and 2 × 2 cm respectively. Third cystic swelling measuring 4 × 4 cm was present in the thoracolumbar region. Skin over the individual swellings were complete. (Fig. 1a, b, c) Anterior fontanelle was soft and open. There was no evidence of other congenital malformations. There were decreased movements in bilateral lower limbs. Muscle tone was normal in all limbs. Anal tone was normal.

**Fig. 2:**
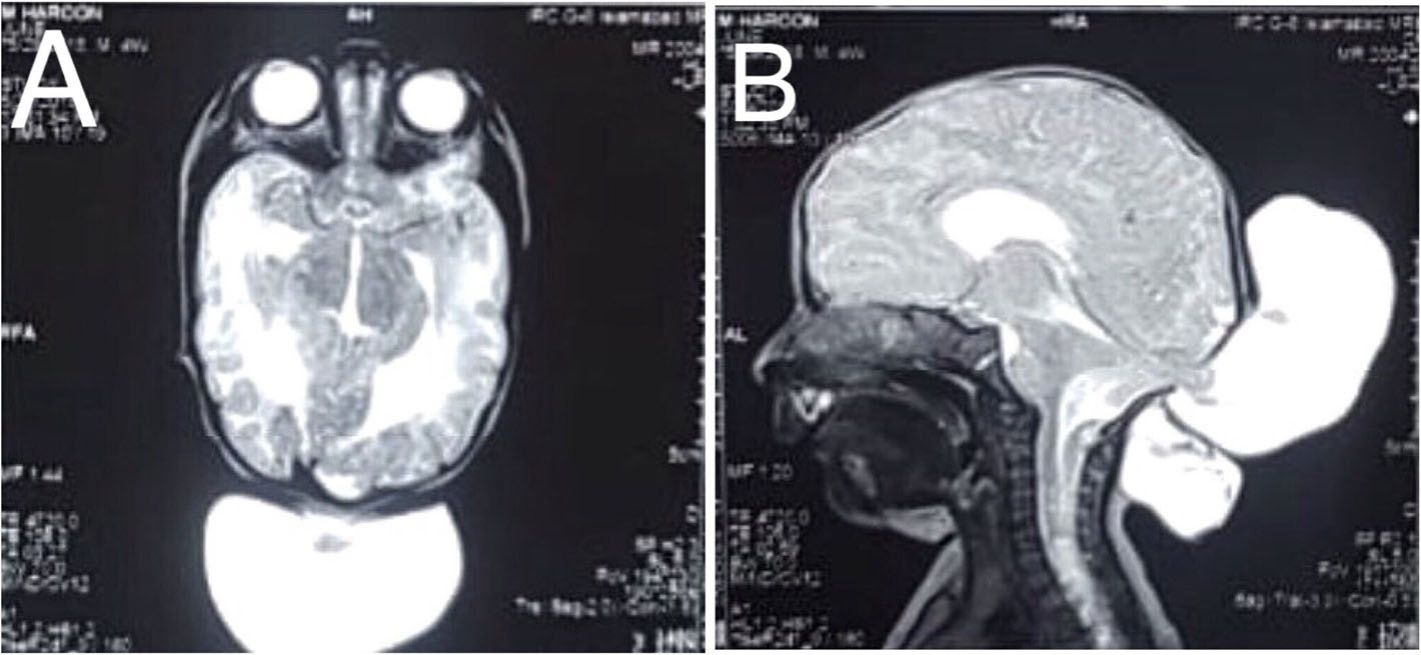
MRI Brain **a** & **b** showing Occipito-cervical meningoencephaloceles

**Fig. 3:**
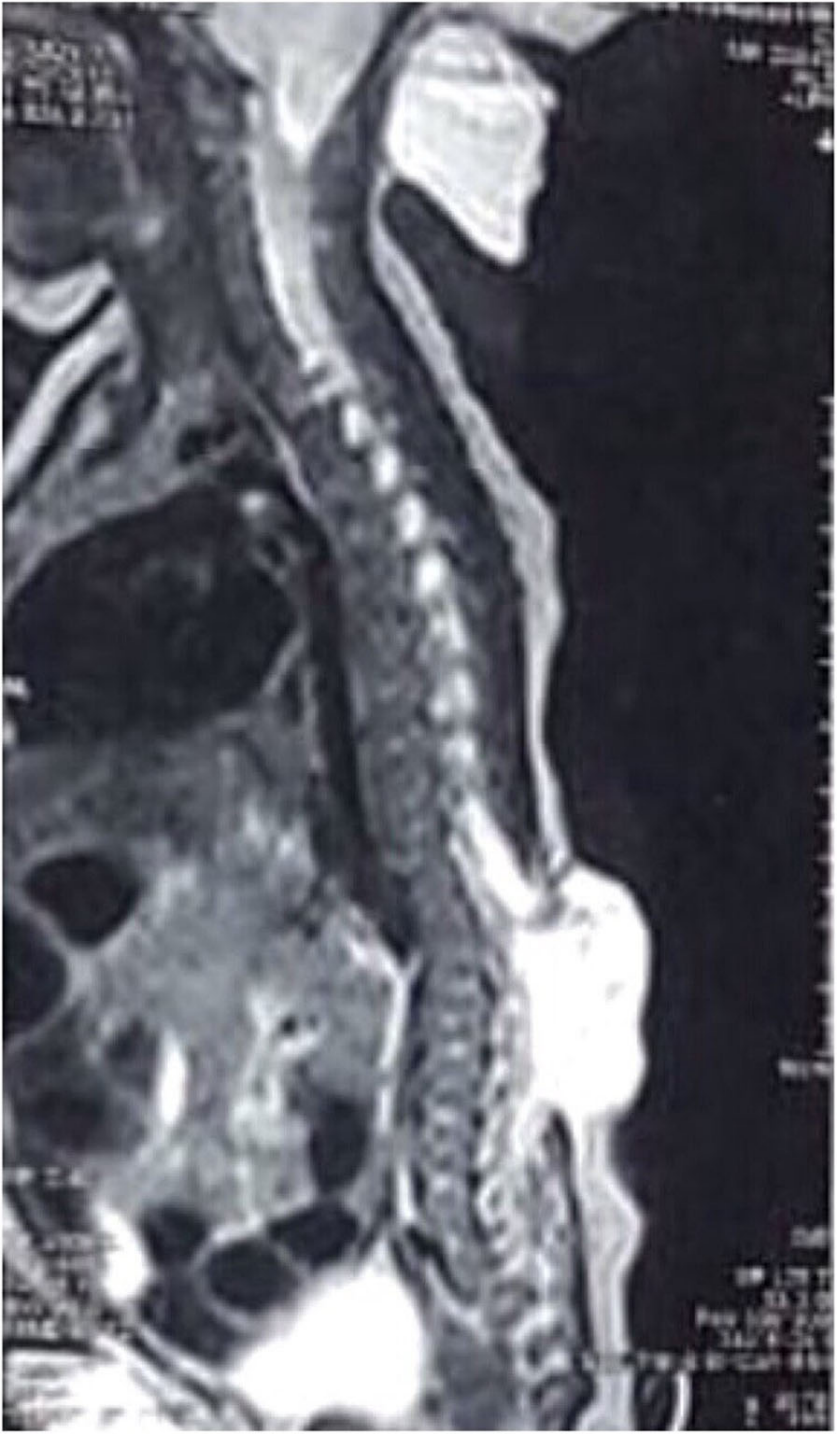
MRI Thoracolumbar spine showing CSF containing sac with neural tissue herniating through a defect in posterior elements of D11 to L1 vertebrae suggestive of thoracolumbar meningomyelocele with syrinx formation in proximal thoracic cord

Magnetic Resonance Imaging (MRI) of the brain revealed a cerebrospinal fluid (CSF) containing sac measuring 9.3 × 4.5 × 5.7 cm with brain tissue herniating through a defect in occipital bone and posterior elements of the upper cervical vertebra in the midline. Bilateral lateral ventricles were mildly dilated. (Fig. 2a, b) With this, the diagnosis of occipito-cervical meningo-encephaloceles with mild hydrocephalus was made for the upper cystic swellings. MRI thoracolumbar spine revealed a CSF containing sac measuring approximately 2 × 3.6 × 3.6 cm herniating through a defect in posterior elements of D11 through L1 vertebrae. An intramedullary hyperintense T2 and hypointense T1 signal were noted opposite to C6 to D11 level suggestive of a syrinx. The diagnosis of thoracolumbar meningomyelocele with syrinx was made. (Fig. 3).

**Fig. 4:**
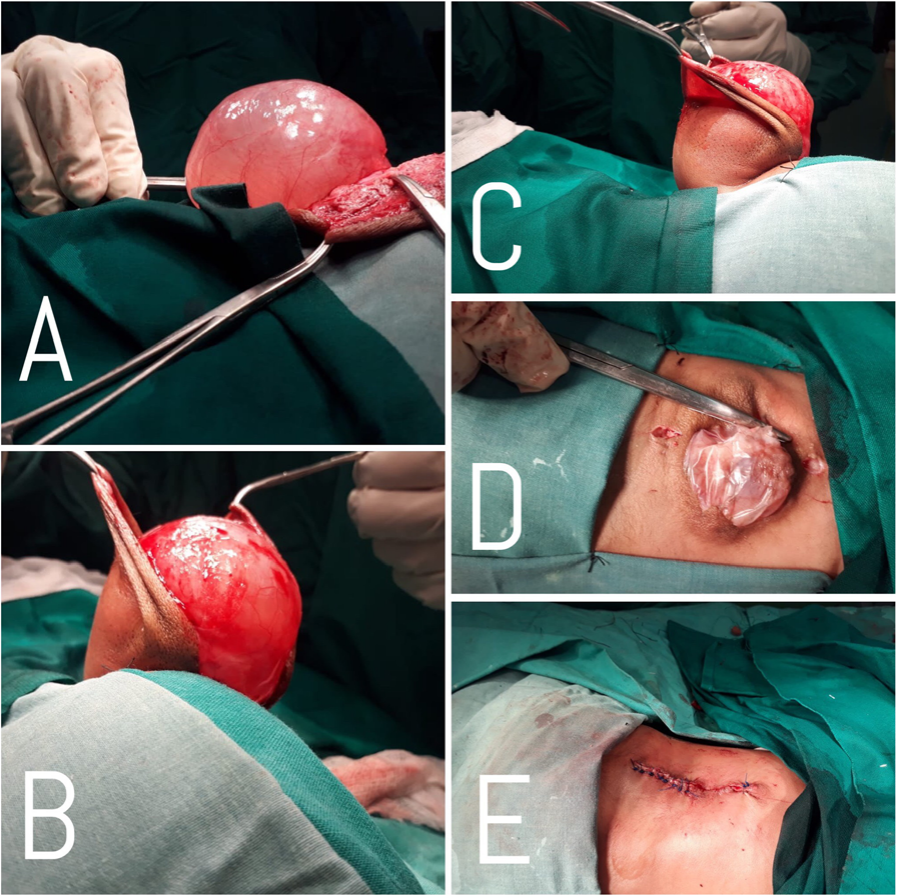
Peroperative **a**, **b** & **c** Occipital Encephalocele, **d** & **e** Thoracolumbar Meningomyelocele

The baby was admitted to the neurosurgery ward with the diagnosis of occipito-cervical meningo-encephaloceles and thoracolumbar meningomyelocele and was planned for excision and repair. Surgery was performed. Both the encephaloceles were repaired first. (Fig. 4a, b, c) Neural tissue was found in upper encephalocele which was pushed back into the cranial cavity and dura was closed with polyglactin 910. Neural tissue was found retracted in the spinal canal while operating on thoracolumbar swelling. (Fig. 4d, e) The neural tissue was separated from the surrounding tissue. Nerve filaments were isolated gently. There was no associated lipoma in the surrounding tissue. Dura was closed with polyglactin 910. Wound was closed in layers with polyglactin 910. The skin was closed with prolene. Valsalva maneuver was performed after repair of dura and skin to check for CSF leak.

Postoperatively, the patient was admitted to the neurosurgical ward and observed for the development of signs of acute hydrocephalus. But the patient remained fine. The patient stayed in the hospital for 1 month. At discharge, the patient was tolerating oral feed. Neurology was the same as in the preoperative period. The patient was followed up again after 1 month and was doing fine.

## Discussion and conclusions

The etiology of neural tube defects is multifactorial involving a complex interaction between gene-gene, gene-environment and gene-nutrients [4–6]. Neurulation involves the proliferation of neuroblasts, differential development of the neuroepithelium and surface ectoderm, formation of a median hinge point in the neural plate, apical constriction of neuroepithelial cells and expansion of the mesoderm and extracellular matrices [7]. Primary neurulation begins at day 18 and completes by day 28 at the S2 region. There is characteristic thickening of the ectoderm from the level of primitive node of hensen caudally to the prechordal plate rostrally forming a slipper shaped structure called neural plate [5]. Secondary neurulation starts at S2 and closes at the caudal region [5].

Many cases of multiple NTDs and severe dysraphism are incompatible with life and death occurs in utero [3]. Hence, most of the earlier information on neurulation was derived from studies in the mouse. Historically, neurulation was thought to start midway along the embryonic body and progress bidirectionally in a zipper like fashion towards cranial and caudal ends, with the closure of anterior and posterior neuropores. Failure of this closure was thought to result in a wide variety of congenital NTDs. While this explained the NTDs present in either poles of the axis, it was not able to explain the presence of intermediate and multiple lesions such as in our case. Multisite closure was hence put forward.

Several patterns of multiple sites of the closure of neural tube have been described in mouse and other species. Four to five (sometimes even six) sites of closure have been proposed and assigned different positions along the rostrocaudal axis. This pattern has been promoted by several authors over the years. Van Allen et al. (1993) proposed five sites of initiation of neural tube closure which proceeded in a sequential manner. Theoretically, it can explain the presence of intermediate and multiple lesion [8]. Srinivas et al. (2008) recently suggested the presence of site 6 at the mid-dorsal level to explain the presence of three meningomyelocele at upper and lower thoracic level and lumbar level [2].

Nakatsu et al., while examining human embryos at stage 10 or earlier observed that closure initiates at three sites: first site at future cervical region, second site at the mesencephalic-rhombencephalic region and third site at the rostral end of neural groove over prosencephalon where anterior neuropore closes [7]. However, observation of human embryos in different stages by O’Rahilly and Muller (2002) found only two de novo fusion sites (α and β) of neural folds and were found to appear in succession. These are at the hindbrain/cervical boundary (rhombencephalic region), called closure 1 in mice and at the rostral extremity of the cranial neural tube (prosencephalic region), called closure 3 in mice. Fusion from rhombencephalic region proceeds bidirectionally (both in rostral and caudal direction) whereas that from prosencephalic region proceeds in caudal direction only. These fusions terminate in two neuropores: anterior and posterior. Further, they found an accessory location of fusion in several embryos in stage 10 and not later. However, its presence was highly variable, both in terms of position and frequency [9].

Mouse closure 2 (between forebrain and mid-brain) does not occur in humans. Interestingly, closure 2 in mice is also variable in location, and is absent in one strain that nevertheless shows successful cranial closure in the majority of embryos [10, 11]. Closure 4 as envisioned by Van Allen et al. does not exist in either mice or humans [12].

The thoracolumbar myelomeningocele is the only defect of primary neurulation in our patient. It results from failure of the closure of caudal neuropore. The occipital and cervical encephalocele are not primary neurulation defects, but rather post-neurulation defects in which the covering layers, the dorsal skeletal elements fail to form and the neural tube herniates outwards. The evidence for this is that an intact neural tube is present in encephalocele. This contrasts with anencephaly, in which an open cranial neural tube is present [12, 13]. Studies from examination of human embryos have depicted two sites of fusion of neural folds and two neuropores. There are no evidences of a pattern of multiple sites of fusion, as described in the mouse, is available in humans [9]. Hence, it would be misleading to speculate about the origins of NTDs, by extrapolating backwards from fetal or postnatal anomalies or constructing embryological details for humans from information derived from other species. Conclusions must be drawn based on proper evidence derived from neurulation studies in humans. Neural tube defects can be explained on the basis of two sites of fusion [13].

Folates and vitamins also have a role in the pathogenesis of neural tube defects along with the role of the gene, environment and their interactions [5, 6]. Folic acid supplementation has been shown to prevent NTD in clinical trials in certain proportion of patients [14]. Maternal conditions (diabetes), maternal infections and maternal exposure to certain drugs like valproate or carbamazepine or anti-folates have shown to result in neural tube defects [5]. Hence, understanding the pathophysiology behind neural tube closure may aid in the development of effective primary prevention strategies and prenatal medical and surgical correction of neural tube defects.

To summarise, the multisite theory seems to offer the best explanation for the development of multiple neural tube defects but observations of human embryos failed to demonstrate more than two closure sites unlike in mouse embryos. The multisite closure theory with more than two sites of fusion, which was forwarded by authors in previous cases of multiple neural tube defects is a mere extrapolation of observation in non-human species that has not been observed in human embryos. There are only two sites of fusion and two neuropores in humans. Encephalocele is a post neurulation defect.

## Data Availability

All data are within the article.

## Abbreviations

CSF: Cerebrospinal Fluid
MMC: Myelomeningocele
MRI: Magnetic Resonance Imaging
NTD: Neural Tube Defect
